# Insights into the respiratory chain and oxidative stress

**DOI:** 10.1042/BSR20171492

**Published:** 2018-10-02

**Authors:** Véronique Larosa, Claire Remacle

**Affiliations:** Genetics and Physiology of Microalgae, UR InBios/Phytosystems, Chemin de la Vallée, 4, University of Liège, Liège 4000, Belgium

**Keywords:** mitochondria, respiratory chain, reactive oxygen species

## Abstract

Reactive oxygen species (ROS) are highly reactive reduced oxygen molecules that result from aerobic metabolism. The common forms are the superoxide anion (O_2_^∙−^) and hydrogen peroxide (H_2_O_2_) and their derived forms, hydroxyl radical (HO∙) and hydroperoxyl radical (HOO∙). Their production sites in mitochondria are reviewed. Even though being highly toxic products, ROS seem important in transducing information from dysfunctional mitochondria. Evidences of signal transduction mediated by ROS in mitochondrial deficiency contexts are then presented in different organisms such as yeast, mammals or photosynthetic organisms.

## Respiratory chain and reactive oxygen species production sites

ATP is the energy carrier compound that is mainly produced in chloroplasts and mitochondria. In both organelles, its production results from oxido-reduction reactions performed by multi-enzymatic complexes located in lipid-bilayer organellar membranes. These reactions are coupled to a proton (H^+^) gradient that is used by the ATP synthase to synthesise ATP from ADP and inorganic phosphate [1].

**Figure 1:**
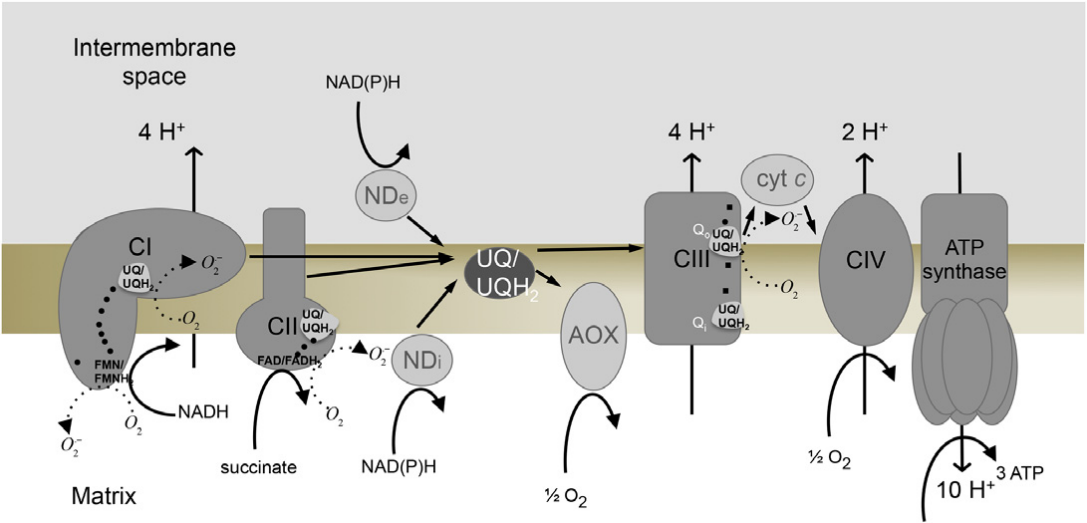
Respiratory chain and ROS production sites Electron transport chain, composed of complex I (CI), complex II (CII), complex III (CIII), complex IV (CIV) and ATP synthase, is presented. Alternative pathways of plant and microorganisms are also shown: alternative NADH dehydrogenase facing the intermembrane space (NDe) or facing the mitochondrial matrix (NDi) and alternative oxidase (AOX). Ubiquinone pool (UQ/UQH_2_). Circles represent Fe–S centres, squares represent haems.

Mitochondrial oxidative process (OXPHOS) comprises four multi-enzymatic respiratory complexes (complexes I–IV) and ATP synthase embedded in the inner mitochondrial membrane. NADH and succinate produced by the Krebs cycle are oxidised by complex I (NADH:ubiquinone oxidoreductase) and complex II (succinate:ubiquinone oxidoreductase) respectively and the electrons are transferred to the ubiquinone pool, leading to the reduction of ubiquinone to ubiquinol inside the mitochondrial membrane. Electrons are then transferred by complex III (ubiquinol:cytochrome *c* oxidoreductase) from ubiquinol to cytochrome *c*, a soluble electron carrier located in the intermembrane space, and from cytochrome *c* to molecular oxygen (O_2_) via complex IV (cytochrome *c* oxidase). The respiratory chain continuously reduces O_2_ into H_2_O in the mitochondrial matrix (Figure 1) but a small quantity of the superoxide anion O_2_^∙−^ is also generated, as a result of monoelectron reduction of O_2_. It has been calculated that less than 0.1% of the electrons passing through the respiratory chain leak on to O_2_ to form superoxide in normal conditions of electron transfer (reviewed in [2]). The respiratory complexes and the other mitochondrial enzymes responsible for such reactive oxygen species (ROS) production are described in this section.

### Complex I

Complex I (EC 1.6.5.3), the first proton-pumping complex, catalyses the reaction.

$$\left[\text{NADH }+\;{\text{H}}^{+}]\;+\;\text{Q }+\;4{\text{ H}}_{\text{matrix}}^{+}\;\to \;{\text{NAD}}^{+}\;+\;{\text{QH}}_{2}\;+\;4{\text{ H}}_{\text{IMS}}^{+}\;(\text{Q },\;\;\text{ubiquinone}\right)$$

#### Subunit composition

In all organisms investigated so far, complex I has an L-shaped form [3] which has been structurally detailed by crystallisation obtained in prokaryotes [4] and eukaryotes such as the fungus *Yarrowia lipolytica* [5] and a few mammals’ species [6–8]. The complex has a molecular weight of approximately 1 MDa and contains more than 40 subunits (reviewed in [9]). Fourteen of these subunits represent the core of the complex as they form the structure of the most simplistic bacterial complex I (type I NADH dehydrogenase) [10,11] and are conserved in all eukaryotic complex I. Among these 14 core subunits, 7 are hydrophobic and usually encoded by the mitochondrial genome (ND1-ND6, ND4L). They form the membrane arm of the complex. The other seven are hydrophilic and encoded by the nucleus (75, 51, 24, 49, 30 kDa, TYKY, and PSST) (bovine nomenclature). They form the matrix arm of the complex and harbour an FMN and eight iron–sulphur (Fe–S) clusters named N3, N1a, N1b, N4, N5, N6a, N6b, and N2 (Table 1). In addition to these core subunits, approximately 30 supernumerary subunits are present in eukaryotic complexes I. Crystallisation of these complexes [5–8] showed that they form a shield around the core subunits and although their role remains unclear, they are thought to stabilise and protect complex I [12].

**Table 1:** The core subunits of complex I and their associated role in electron or proton transfer as summarised in [11]

Core subunits (bovine nomenclature)	Role
51 kDa	Binding of FMN - N3 - NADH
24 kDa	Binding of N1a
75 kDa	Binding of N1b, N4, N5
PSST	Binding of N2
TYKY	Binding of N6a, N6b
49 kDa-PSST-ND3-ND1	Binding of quinone
ND2, ND4, ND6, ND1-ND6-ND4L	Proton translocation

#### Electron transfer pathway

The substrate NADH binds to the 51-kDa subunit near the FMN moiety. Two electrons are extracted from NADH and transferred to the FMN molecule in a binding pocket detailed in [11] for bacterial complex I. The FMN serves as the first electron acceptor from NADH; the N3, N1b, N4, N5, N6a, and N6b serve as a path for electron transport [13,14] (Figure 1). The N2 cluster serves as final electron acceptor and catalyses electron transfer to the ubiquinone molecule. An eighth Fe–S cluster, named N1a, located near the FMN, is thought to serve as an electron store meant to avoid an excessive ROS production [5].

The quinone-binding site is formed by the 49 kDa-PSST-ND3-ND1 subunits in bacteria [11]. This module allows electron transfer to the ubiquinone through the N6a and N6b Fe–S clusters located on the TYKY subunit and through N2 reaction centre cluster.

#### Proton translocation pathway

Four proton channels are located in the membrane domain and participate in proton pumping: three in antiporter-like subunits (ND2, ND4 and ND5) and one at the interface with the hydrophilic domain (ND1-ND6-ND4L) [11]. The proton-pumping channels through the membrane domain are distant from the redox-active groups mediating electron transfer through the hydrophilic domain. The current model for proton pumping proposes that the stabilisation of a negatively charged ubiquinone at the ubiquinone-binding site would be responsible for a conformational change causing energy transmission to the membrane arm and resulting in proton pumping [4,11,14,15].

#### *ROS production* (reviewed in [2])

ROS are produced in two situations: (i) when electrons are backed up in the chain of Fe–S clusters. This happens when NADH is present and the downstream chain is inhibited by complex I inhibitors that bind to the quinone-binding site or by inhibitors of complex III or complex IV; and (ii) in conditions of reverse electron transfer, when electrons are going backwards from complex II via ubiquinone to complex I. In the ‘forward’ mode, the electrons delivered by NADH produce ROS at the FMN cofactor; in the ‘reverse mode’, superoxide is generated from a semi-ubiquinone at the quinone-binding site. There are also experimental evidences that this mode could also produce superoxide at the FMN moiety [16,17] but this is still to be investigated. The reverse electron transfer requires a high membrane potential or proton-motive force.

### Complex III

Complex III (EC 1.10.2.2), the second proton-pumping complex, catalyses the reaction.

$$2{\text{QH}}_{2}\;+\;2\text{ ferricytochrome }c\;+\;2{\text{H}}_{\text{Matrix}}^{+}\;\to \;{\text{QH}}_{2}\;+\;2\text{ ferrocytochrome }c\;+\;4{\text{H}}_{\text{IMS}}^{+}$$

#### Subunit composition

Complex III always functions under a dimeric form, with a size of ≈500 kDa. The monomeric form of complex III contains ten to eleven subunits: three core subunits of prokaryotic origin, cytochrome *b*—cytochrome *c_1_*—Rieske iron–sulphur protein, and eight supernumerary subunits. Cytochrome *b* contains the ubiquinol oxidation centre (Q_o_) and ubiquinone reduction centre (Q_i_), separated by two *b*-type haems. One haem (*b*_L_) is close to the Q_o_ site and has a low redox potential, and the other haem (*b*_H_) is close to the Q_i_ site and has a high potential haem [17]. Cytochrome *c_1_* contains a *c*-type haem.

#### Proton-motive Q cycle and electron transfer

The molecular mechanism that drives H^+^ pumping of complex III has been first proposed by Mitchell [18] and supported by numerous studies (reviewed in [2]). In short, the proton-motive Q cycle starts with the oxidation of ubiquinol at the Q_o_ site, and the release of two H^+^ at the intermembrane space. One electron is transferred successively to the Fe–S cluster of the Rieske protein, to cytochrome *c_1_*, and then to soluble cytochrome *c*. The other electron is successively transferred to the haem *b*_L_ of cytochrome *b*, to haem *b*_H_ and finally to a ubiquinone bound to the Q_i_ site, which is reduced to a stabilised semiquinone species. In the second round of the cycle, the electron entering the Fe–S cluster of the Rieske protein reduces a second cytochrome *c* and *c_1_*, which is accompanied by two other H^+^ released at the intermembrane space. The electron entering the low-potential chain reduces the semiquinone in the Q_i_ site to ubiquinol. This is accompanied by the uptake of two protons from the negative matrix side of the mitochondrial membrane. Thus there is a net translocation of 2H^+^/2e^−^. The key component of the Q cycle is the correct routing of the two electrons resulting from ubiquinol oxidation to either the Fe–S cluster of the Rieske protein or to the *b*_L_ haem of cytochrome *b* [19,20]. The flexibility of the globular domain of the Rieske protein seems to be one of the main elements for the bifurcation of the electrons. Other components implicated in this correct routing could be the binding of ubiquinol into the Q_o_ site that would trigger conformational changes, maybe due to the widening of Q_o_ to accept the substrate [21].

#### ROS production

Superoxide production is formed at the ubiquinol oxidation centre of complex III (Q_o_) and induced by adding antimycin A, a specific inhibitor of the Q_i_ site of the complex. In addition, a high membrane potential can also enhance the superoxide production at the Q_o_ site (reviewed in [2,22]). The redox state of the ubiquinone pool is another factor responsible for superoxide production in the presence of antimycin A [22]. Superoxide production could be explained because in all these conditions, electrons are backed up in cytochrome *b*, which leads to an accumulation of semiquinone radical at the Q_o_ site, which can transfer its electron to oxygen forming superoxide. Other authors propose that superoxide production at the Q_o_ site occurs by reverse electron transfer from reduced haem *b*_L_ directly on to molecular oxygen and that oxidised ubiquinone serves as a redox mediator (reviewed in [2,22]).

### Complex II

Complex II (1.3.5.1) catalyses the reversible conversion of succinate into fumarate. It is the only membrane-bound enzyme of the Krebs cycle and does not contribute to the formation of a proton gradient.

$$\text{succinate }+\;\text{Q }↔\;\text{fumarate }+\;{\text{QH}}_{2}$$

#### Subunit composition and electron transfer

The size of this small multiprotein complex is ≈110 kDa. Four subunits are found in most eukaryotes (reviewed in [9]). The two matrix subunits, Sdh1 and Sdh2, are anchored to the membrane by the Sdh3 and Sdh4 membrane subunits. The Sdh1 subunit contains a covalently attached FAD in the dicarboxylate binding site, where succinate is oxidised into fumarate [23]. The Sdh2 subunit bears three Fe–S centres. Sdh3 and Sdh4 harbour two ubiquinone reduction sites and a *b*-type haem at the interface of both subunits. The enzyme catalyses the oxidation of succinate to fumarate and transfers the two electrons of the reaction to ubiquinone-bound membrane via four prosthetic groups (the FAD cofactor, and the three Fe–S centres). Only one of the ubiquinone reduction sites is used. In addition, the *b*-type haem is located off pathway from the electron transferring cofactors and does contribute to the electron transfer between succinate and quinone bindings [23].

#### ROS production

ROS production by complex II is by far less studied than that of complexes I and III. It has been shown that superoxide is produced at the FAD site of the complex in the direct and in the reverse reaction, when the electrons are provided from the reduced ubiquinone pool [24]. The quinone-binding site would not be a site for superoxide production in the wild-type enzyme (reviewed in [23]).

Additional sites of ROS production are present in mitochondria. Some enzymes produce ROS at their flavin site (the 2-oxoacid dehydrogenase complexes). Other enzymes produce ROS at their Q-binding site (mitochondrial 3-phosphate dehydrogenase, the short electron transfer chain composed of electron transfer flavoprotein (ETF) and the ETF:ubiquinone oxidoreductase (ETF:QO), dihydroorotate dehydrogenase) (reviewed in [25,26]).

### 2-oxoacid dehydrogenase complexes

2-oxoacid dehydrogenases complexes are NADH/NAD^+^-linked enzymes, which bind flavin molecules and contribute to superoxide/H_2_O_2_ production in the matrix of mitochondria at their flavin site. They oxidise different substrates (2-oxoglutarate, pyruvate, branched-chain 2-oxoacids or 2-oxoadipate) to the corresponding acyl-CoA and reduce NAD^+^ into NADH + H^+^. They have similar subunit composition (E1, E2 and E3) (see below), these proteins being encoded by multigene families. Their superoxide/H_2_O_2_ production has been studied and compared in isolated rat mitochondria at the redox potential of NADH/NAD^+^ by [27]. These authors showed that the maximum ROS production follows the range 2-oxoglutarate dehydrogenase > pyruvate dehydrogenase > branched chain 2-oxoacid dehydrogenase > complex I, leading to propose that H_2_O_2_ production by the 2-oxoglutarate dehydrogenase may be considerable and possibly previously misattributed to complex I.

#### 2-oxoglutarate dehydrogenase complex

The 2-oxoglutarate dehydrogenase complex (OGDH) complex catalyses the overall conversion of 2-oxoglutarate into succinyl-CoA, CO_2_ and NADH in the Krebs cycle. It contains three components. The E1 component is an oxoglutarate decarboxylase that contains thiamine pyrophosphate as cofactor. The E2 component is a dihydrolipoyl succinyltransferase that contains lipoic acid and coenzyme A as cofactors. The E3 component of the enzyme (dihydrolipoyl dehydrogenase) binds FAD and NAD^+^ and produces superoxide/H_2_O_2_ at the flavin site when NAD^+^ is limiting (high NADH/NAD^+^ ratio) [27].

#### Pyruvate dehydrogenase complex

The pyruvate dehydrogenase (PDH) complex catalyses the overall conversion of pyruvate into acetyl-CoA, CO_2_ and NADH in the mitochondrial matrix. Similar to the OGDH complex, it is composed of three components, E1, E2 and E3. E1 is a pyruvate dehydrogenase containing thiamine pyrophosphate as cofactor, E2 is a dihydrolipoyl transacetylase containing lipoic acid and coenzyme A as cofactors, E3 is dihydrolipoyl dehydrogenase that binds FAD and NAD^+^. Similar to OGDH, superoxide/H_2_O_2_ is produced at the flavin site when NAD^+^ is limiting [27].

#### Branched-chain 2-oxoacid dehydrogenase complex

This complex catalyses the overall conversion of branched chain 2-oxoacids produced by the catabolism of valine, isoleucine and leucine to acyl-CoA, CO_2_ and NADH. It contains three enzymatic components: branched-chain 2-oxoacid decarboxylase (E1), lipoamide acyltransferase (E2) and lipoamide dehydrogenase (E3). It produces superoxide/H_2_O_2_ at high NADH/NAD^+^ ratio at the flavin site [27]. Branched-chain 2-oxoacid dehydrogenase complex (BCKDH) has been found in plants [28] but to our knowledge, no report about superoxide production has been published yet.

#### 2-oxoadipate dehydrogenase complex

This complex catalyses the overall conversion of 2-oxoadipate into glutaryl-CoA, CO_2_ and NADH. 2-oxoadipate is produced by the catabolism of tryptophan, lysine and hydroxylysin [29]. Rat skeletal muscle mitochondria produce superoxide/H_2_O_2_ at high NADH/NAD^+^ ratio at the flavin site [29].

### Mitochondrial glycerol-3-phosphate dehydrogenase

This enzyme is a part of the glycerol-3-phosphate (G-3-P) shuttle that channels cytosolic reducing equivalent to mitochondria for respiration through oxidoreduction of G-3-P in different mammalian tissues, yeasts and higher plants [30,31]. This shuttle uses two GDPHs: a cytoplasmic NADH-coupled enzyme reducing dihydroxyacetone phosphate to G-3-P and an FAD-linked ubiquinone oxidoreductase enzyme at the outer face of the inner mitochondrial membrane re-oxidising G-3-P and feeding the electrons directly to the ubiquinone pool. The mitochondrial GDPH monomer has a size of 74 kDa in mammals and could be active under dimeric or even multimeric forms (reviewed in [31]). Its Q-binding pocket has been suggested to be the major site of superoxide generation in different mammal tissues [32,33].

### Mitochondrial dihydroorotate dehydrogenase

This enzyme catalyses the ubiquinone-mediated oxidation of dihydroorotate to orotate, at the outer face of the inner mitochondrial membrane and constitutes the fourth enzymatic step of pyrimidine synthesis. In mitochondria from rat skeletal muscle, this FMN-linked ubiquinone enzyme produces superoxide at the ubiquinone site [34]. A dihydroorotate dehydrogenase (DHDOH) has also been identified in plant mitochondria [28] but its involvement in superoxide/H_2_O_2_ production has not been reported yet.

### ETF:QO (reviewed in [35])

ETF:QO enzymes transfer their electrons to ubiquinone via a short electron transfer chain composed of ETF and ETF:QO. The electrons are generated by β-oxidation of fatty acids at the level of the acyl-CoA dehydrogenases. ETF:QO has been purified from pig liver mitochondria: it is a monomer of 68 kDa associated with the inner mitochondrial membrane and contains FAD and a single Fe–S cluster as cofactors [35]. Superoxide formation by ETF:QO has been proposed to be associated with impaired electron transfer from flavin to the Q-binding pocket in mammal tissues [35]. The ETF/ETF:QO electron transfer chain has also been reported in plants [28].

### A more oxidant destiny of O_2_^∙−^ and H_2_O_2_

Once O_2_^∙−^ and H_2_O_2_ are generated, other ROS molecules could arise from reactions with transition metal residues. Indeed, hydroxyl radical (HO∙) could be formed through iron redox cycling by Fenton reaction, where free iron (Fe^2+^) reacts with H_2_O_2_, and by the Haber–Weiss reaction that results in the production of Fe^2+^ when superoxide reacts with ferric iron (Fe^3+^) [36]. In addition to the iron redox cycling, a number of other transition metals including Cu, Ni, or Co could be responsible for HO∙ formation in living cells [36]. The protonated form of H_2_O_2_, the hydroperoxyl radical (HOO∙), being another ROS, can also react with redox active metals such as Fe or Cu to further generate HO∙ through the above-described reaction [36].

## ROS damage and detoxification

It is assumed that the largest fraction of ROS has a mitochondrial origin [37], although they can also be produced in each other’s compartment that includes proteins or molecule with a sufficiently high redox potential to excite or donate an electron to atmospheric oxygen [38]. The chemical nature of the substrates fueling the respiratory chain, the amplitude of the membrane potential in mitochondria (ΔΨm), the pH of the matrix, and the oxygen tension in the surroundings are important factors controlling ROS production in mitochondria [39,40], this control having to be tight for such molecules considered as toxic by-products. Indeed, ROS can damage cells in many ways and give rise to fast, barrier-less and non-selective oxidation steps, being responsible for a severe insult of both organic and inorganic matter exposed to ‘oxidative stress’ [41,42]. Protein oxidation mostly results in the formation of carbonyl groups (ketone and aldehydes) [43]. HO∙ and HOO∙ are responsible for the oxidation of lipids, thus leading to impairment of membrane function [44–46]. DNA bases can be modified by Fenton gated oxidative stress [47,48].

Beside the fact that mitochondrial H_2_O_2_ can cross the membrane and serve as signalling molecule (see point 3), nature has evolved a complex enzymatic machinery to control the risk of so-called ‘oxygen toxicity’ paradox [41,42]. The primary line of defence is a panel of proteins that remove ROS or that act as sequestering metal ions that are reviewed below. Briefly, superoxide production can be detoxified into H_2_O_2_ in a reaction catalysed by superoxide dismutase (SOD). Afterwards, H_2_O_2_ can be removed by antioxidant enzymes such as catalase (CAT) and peroxidase, which convert H_2_O_2_ into water.

### SOD

SODs are metalloenzymes. The superoxide dismutation reaction starts with the reduction of the metal centre and is followed by its reoxidation by superoxide radical anion. It is dependent on two protons per redox cycle [49]. SODs are present in prokaryotes and in eukaryotes, found in monomeric, dimeric or tetrameric conformation and classified on the basis of their metal cofactor. So far, four types are identified: manganese co-factored (MnSOD), iron co-factored (FeSOD), copper/zinc co-factored (Cu/ZnSOD) and nickel co-factored (NiSOD) (reviewed in [49–51]). Superoxide production by complex III at the intermembrane space can be detoxified into H_2_O_2_ in a reaction catalysed by Zn/CuSOD, while superoxide produced in the matrix side by complex I can be detoxified into H_2_O_2_ by MnSOD [52]. Indeed, except in the diatom *Thalassiosira pseudonana* where an MnSOD has been identified in chloroplasts [53], it is generally assumed that MnSODs are found in mitochondria of eukaryotes.

### CAT

CATs can be of two types Mn-CAT and haem-CAT [54]. They have one of the highest turnover rates of all enzymes, converting approximately 6 million molecules of H_2_O_2_ into H_2_O and O_2_ per minute and per molecule of CAT [55]. While the hexameric Mn-CATs only exist in prokaryotes, tetrameric haem-CATs, which contain one molecule of haem per subunit, are found in a much wider range of organisms. In eukaryotes, the predominant form is the tetrameric haem form and is mainly localised in peroxisomes, where hydrogen peroxide is produced by the acyl-CoA oxidase of the fatty acid β-oxidation pathway or by other oxidases [54], leaving glutathione peroxidase (GPX) as the major scavenger in mitochondria to deal with H_2_O_2_ reduction. In higher plants, among the numerous CAT isoforms identified that could be highly expressed in specific cell types, some of them seem to be found in mitochondria but studies are ambiguous. Indeed, proteomic analysis of highly purified mitochondria from *Arabidopsis* cells identified CAT2 and CAT3 peptide sequences [56]. This finding was interpreted with some caution since CAT activity, used as a marker for peroxisomal contamination, showed a progressive decline throughout the mitochondrial purification procedure, along with the plastid marker enzyme, alkaline pyrophosphatase [56]. In yeast, peroxisomal CAT was co-localised to mitochondria in a manner that depended on nutritional conditions [57]. Dual peroxisomal/mitochondrial targeting of CATs cannot yet be ruled out. However, to date there has been no demonstrations of CAT import into mitochondria using either *in vitro* or *in vivo* approaches, and it is possible that contamination may account for reports of CAT activity in this organelle [58]. The same uncertainty exists in algae such as in the green alga *Chlamydomonas reinhardtii* where there is only one isoform of CAT (CAT2, [59]). This enzyme has been detected in isolated mitochondria [60] and by proteomic analyses of purified mitochondria [61] although the targeting prediction tool (Predalgo, [62]) does not identify any putative mitochondrial targeting in the amino acid sequence of the protein. In addition, CAT2 presents a weak PTS1 signal (peroxisomal) at the C-terminus (VEL) based on the consensus sequences established for the peroxisomal targeting in higher plants by [63].

Recently a synthetic ‘dizyme’ combining SOD and CAT functional activities has been designed to enable a detoxification cascade from O_2_^∙−^ into H_2_O and O_2_. This ‘dizyme’ has been shown to prevent H_2_O_2_ accumulation in the green alga *Chlamydomonas reinhardtii*, cultivated under high light illumination conditions [42].

### Peroxidase

Peroxidases catalyse the reduction of H_2_O_2_ into water using reductants that give the name of the peroxidase, e.g. ascorbate peroxidase, GPX and thioredoxin peroxidase. These enzymes are present in practically all subcellular compartments and an organelle has generally more than one system to scavenge ROS [64,65]. Ascorbate peroxidases are only present in photosynthetic organisms where they play an important role for detoxification of H_2_O_2_ produced by photosystem I in the chloroplasts (reviewed by [66]). Eight isoforms of GPX are found in the cytoplasm and mitochondria in mammals, which are dependent on selenium for their antioxidant function [67]. Oxidised glutathione (GSSH) is reduced by its corresponding glutathione reductase, which uses NADPH as substrate. In mitochondria, NADPH can be kept reduced by the activity of H^+^-transhydrogenases [52]. Thioredoxin peroxidases are multigene families in eukaryotes with some of the isoforms found in mitochondria [68]. Similar to the system described for the GPX, oxidised thioredoxin (TrxS^−^) is reduced by its corresponding thioredoxin reductase, using NADPH as substrate and H^+^-transhydrogenases can keep the NADPH reduced [52].

### Alternative enzymes of the respiratory chain

In plants and many microorganisms, besides the main respiratory enzymes—complexes I–IV— alternative enzymes which do not contribute to the proton gradient are present (Figure 1): NAD(P)H dehydrogenases (type II NAD(P)H dehydrogenases) and alternative oxidases (AOXs). These enzymes are not relevant in standard physiological conditions since they are not coupled to ATP synthesis and thus reduce the energy efficiency of respiration. However, they are useful when there is a need to uncouple electron transfer and ATP production, thereby avoiding ROS formation, a situation that is described to occur in many conditions such as the stationary growth phase in microorganisms [69] or under different biotic and abiotic stresses (reviewed in [70]).

NAD(P)H dehydrogenases transfer electrons from NADH to the ubiquinone pool, facing either the intermembrane space or the mitochondrial matrix. The crystal structure of one of them from yeast, located on the matrix side, has been obtained in 2012 [71]: the structure reveals two ubiquinone-binding pockets, and the FAD- and NADH-binding sites. The enzyme is functional under a dimeric form. In the green microalga *C. reinhardtii*, inactivation of NDA1, located at the inner side of the mitochondrial membrane does not lead to any clear physiological defect except if associated with a complex I deficiency [72]. AOXs are homodimeric, and the monomeric unit has a size of approximately 40 kDa. They contain a covalently bound diiron centre that catalyses the four-electron reduction of dioxygen to water by ubiquinol [73]. The role of AOX in the control of ROS and their signalling in plants has been recently discussed in [74]. The presence of these two types of enzymes allows for better survival of *C. reinhardtii* mutants affected in complex I, III or IV and partly explains why isolated mutants with deficient OXPHOS complexes are viable in the microalga *Chlamydomonas* [9].

## ROS signalling

ROS could potentially be considered as an essential factor in cell-signalling processes thanks to the fact that they are produced in different sites, are very stable and can potentially diffuse through appreciable distances or travel across membranes [38,75]. These cell-signalling processes termed as redox biology, in which ROS act as signal transducers, appear early in the evolution and are proposed to allow adaptation of organisms to oxidative conditions [76]. So far, an increasing number of studies showed that «waves of oxidative compounds» as well as antioxidants reactions, activate gene expressions or responses in a variety of phylogenetically different organisms [77,78] and in response to a lot of environmental challenges.

### Mode of action

The mode of action of ROS signalling is about transducing the signal mainly via interaction with cysteine (Cys) residues of proteins [79–81] and like this modifying protein functions. Indeed, the cysteine thiol group (Cys–SH) represents the oxidation state −2 of the sulphur atom, the fully reduced form. This sulphur atom becomes a reactive nucleophile in the deprotonated form [82]. In fact, H_2_O_2_ could interact with Cys thiolate anions (Cys–S), at physiological pH, leading to their oxidisation to sulphenic form (Cys–SOH). In this condition, the oxidation state of the sulphur atom rises to 0. This allows Cys–SH to undergo several post-translational modifications, thanks to structural changes within the protein. This happens in plant or in animal cells as reviewed in [78]. Cys–SOH is highly reactive and, under major ROS excess, can be overoxidised to sulphinic acid (Cys–SO_2_H) and sulphonic acid (Cys–SO_3_H) [78]. Although these overoxidations have been regarded as irreversible modifications in the past, sulphiredoxin enzymes have been shown to reduce SO_2_H through an ATP-dependent reaction [83–86]. These redox-derived changes in protein function can affect transcription, phosphorylation and other important signalling events, and/or alter metabolic fluxes and reactions in the cell by altering enzymatic properties of the proteins [79–81].

Additionaly, Cys–SOH can also react with a free thiol on the same protein, on other proteins, or with low-molecular weight thiols, such as glutathione. Indeed, tripeptide glutathione (GSH) could form disulphides either with proteinaceous Cys or with itself. GSH, being the most abundant and widely distributed low-molecular thiol compound of the cell, is found in most subcellular compartments mainly in its reduced form and transiently accumulating as oxidised disulphide (GSSG) under stress conditions [87].

In this way, mitochondrial ROS could induce communication with the nucleus, the so-called retrograde signalling pathway that was originally discovered as a mechanism initiated by mitochondrial dysfunction in yeast [88]. Such ROS retrograde signalling could then involve an essential cellular adaptation mechanism by which dysfunctional mitochondria can transmit signals to a nuclear level in order to adapt the metabolic machinery to survive [89]. In fact, in normal conditions mitochondria do not export ROS but are preferentially a sink for them [90]. However, under stress conditions the capacity of antioxidant systems can be exhausted and the direction of ROS flux can be reverted. For instance, an increase in cytosolic [Ca^2+^] transforms yeast mitochondria into a major source of ROS, by the fact of a burst in generation of ROS by complex III [40].

### Calcium signalling

Interactions between Ca^2+^ and ROS are considered as bidirectional: ROS can regulate cellular calcium signalling, while calcium signalling is essential for ROS production [91]. Indeed, as cited above ΔΨm could control ROS production. This was shown for example by the correlation between the use of uncouplers and the reduction in ROS production [40]. Concerning specificity of Ca^2+^, when ΔΨm is high, Ca^2+^ uptake results in a decreased ROS generation, while if ΔΨm is depolarised, ROS generation is stimulated or not influenced by Ca^2+^ [92]. Additionally, the explained above redox modification of disulphide bonds is known to affect the structure and function of ion regulatory proteins including ion channels, pumps and transporters. This includes cardiac calcium channel and transporters [93] that are the mainly studied topics so far. ROS and Ca^2+^ are also correlated by their common point: ATP. The interconnection between ATP, ROS and Ca^2+^ was called the mitochondrial love-hate triangle by Brookes et al. (2004) [94]: Ca^2+^ promotes ATP synthesis by stimulating Krebs cycle enzymes and oxidative phosphorylation. As the whole mitochondria work faster and consume more O_2_, ROS levels increase, because of an increased respiratory electron leakage, which could lead to the negative feedback from respiratory chain and a decrease in ATP production.

## Mitochondrial deficiencies and their impact through ROS signalling

### Impact of mitochondrial ROS on respiratory chain subunits

In mammals, ROS could mediate the regulation of nuclear components of the above-described respiratory chain. For example, mitochondrial ROS were shown to regulate a nuclear miRNA component, miR-663 in tumor cells, that specifically control the expression of nuclear-encoded structural subunits or assembly factors of I, II, III and IV complexes [95]. Moreover, ROS can activate a tyrosine kinase, Fgr, allowing the phosphorylation of the SDH subunits of complex II, which allows adjusting the level of complex I in order to optimise the NADH/FADH_2_ electron use in the respiratory chain [96]. In addition, ROS generated by complex I or III specifically react with distinct subsets of proteins in isolated mitochondria from rat heart [97]. Indeed, redox fluorescence difference gel electrophoresis analysis showed that proteins involved in β-oxidation and fatty acid import are preferentially complex III redox-sensitive targets while proteins of the Krebs cycle are preferentially complex I redox targets. It is also proposed that H_2_O_2_ formed at the level of complex III could have a direct feedback on complex I enzyme [2] or that ROS at complex I have an impact on complex II components [97]. Moreover, lipid-derived reactive species formed in mitochondria could react with mitochondrial components resulting in mitochondrial dysfunction or in the regulation of cell function [98–100]. Interestingly, nuclear complex I proteins appear as a specific target in a signalling event derived from mitochondrial polyunsaturated fatty acids lipoperoxidation promoted by HO∙ [101,102].

### Impaired respiration/mitochondrial function and signalling

In a respiratory altered background, such as when in yeast, complex III is inhibited by myxothiazol, the signal is relayed to the cell by the modulation of transcription factors known to be involved in oxidative stress response, such as Yap1 [103], a basic leucine zipper transcription factor that is a key cytosolic H_2_O_2_ sensor [104]. Indeed, H_2_O_2_ may activate Yap1 by oxidising its Cys–SH in Cys–SOH by the thiol peroxidase Orp1. Consequently, a Cys–SOH chaperone, Ybp1, brings together Orp1 and Yap1 into a ternary complex that selectively activates condensation of the Orp1 to provide specificity in the transfer of oxidising equivalents by a reactive sulphenic acid species [105]. In yeast, respiratory complex III deficiencies could then be compensated by a ROS modulation of the Yap1 signalling process [106].

Concerning mammals, alterations of mitochondrial systems have long been documented in tumours, and the disruption of the mitochondrial retrograde signalling seems implicated in this process. Evidence seems to correlate the influence of ROS, and specifically when formed in a complex III deficiency background, on the nuclear expression of oncogenes/tumour suppressors (such as *APC* gene) and of some kinases such as MAPK/ERK that can activate tumorigenesis [107]. In contrast with deficiencies, an overexpression of a complex I structural subunit, the ND3 subunit, also highlights the role of ROS signalling in tumour formation [108]. Indeed, such overexpression involves a ROS-mediated reduction in a glycolytic enzyme, the pyruvate kinase M2, by its carbonylation, this being a pro-cancerous feature [108].

The pathophysiology of cancer in association with mtDNA variations is suggested to be a manifestation of elevated ROS, reported as a mitogenic mediator and as an inducer of apoptosis [38,109,110]. In fact, as the majority of the mtDNA encodes for proteins of the mitochondrial respiratory chain and as mtDNA could potentially be targeted by oxidation, a correlation between ROS formation and mtDNA mutations exists. Indeed, in two different knockout mice models with increased mitochondrial ROS due to MnSOD and aldehyde dehydrogenase deficiencies, correlation between mitochondrial ROS formation and oxidative mtDNA lesions is increasing with age [111]. Concerning a cell signalling related to mtDNA oxidation, the case of mitochondrial transcription factor A (TFAM) is interesting. TFAM, being an mtDNA-binding protein and the major regulator of mtDNA copy number in mammalian models [112], seems to have a regulatory mode over ROS production and calcium. Indeed, TFAM allows the stabilisation of a regulatory complex of mtDNA depending on an increase in ROS and calcium conditions. When mitochondria become dysfunctional such as in failing hearts, TFAM level initially rises as a compensatory mechanism, but it progressively decreases as calcium mishandling and ROS production increase, as observed in later stages of heart failure, TFAM being lost in dysfunctional mitochondria [113].

The mitochondrial and ROS signalling process in photosynthetic organisms is also of high interest because it seems to be crucial for the adaptation to environmental conditions and is linked to biotic and abiotic stresses (reviewed in [114]). In a complex I mutant of *Arabidopsis thaliana*, proteome analysis showed reorganisation of both cellular respiration and photosynthesis, which is proposed to be responsible for the increase in ROS and stress defence system [115]. Induction of the expression of a twin cysteine protein (At12Cys) in this type of mutant has been proposed to be responsible for modification of cytosolic, chloroplastidic and mitochondrial functions [116]. In an *A. thaliana* complex II mutant, ROS production in roots and leaves are lowered in response to stresses such as salicylic acid or bacterial infection, suggesting a role of complex II in plant stress and defence stress responses through mitochondrial ROS signalling [117]. Using a forward genetic screen to characterise regulators of *AOX1* expression in *A. thaliana*, Ng et al. [118] found a transcription factor of the NAC family, ANAC017, which is bound to the endoplasmic reticulum and released upon mitochondrial perturbation to initiate the mitochondrial retrograde response.

The production of ROS has also been investigated for some of the respiratory mutants of the microalga *C. reinhardtii*: in complex I mutants, H_2_O_2_ production is not modified in moderate light compared with control strains [119] and ROS detoxification enzymes are lowered [120]; in mutants affected in the COX3 subunit of complex IV, a 60% decrease in H_2_O_2_ production after short exposure (12 h) to darkness is found compared with wild-type [121].

Chloroplasts are other sources of ROS in photosynthetic organisms and these organelles play a major role in ROS production. In the green microalga *C. reinhardtii* grown in high light, ROS production seems to be mainly caused by the chloroplast since the increase in H_2_O_2_ production is the same in mitochondrial mutants (such as complex I mutants or AOX mutants) as in control strains [122]. Therefore, the mitochondrial ROS signalling does not seem to be relevant in high light in these mutants. However, it seems implicated in other growth conditions, as shown by Murik et al. [123]. These authors analysed the response of a complex III mutant to oxidative stress and programmed cell death in control light and brought evidence that it was different from its control strain, which suggests that respiratory deficient mutants could be interesting tools to study mitochondrial and ROS signalling in microalgae.

## Abbreviations

AOX: alternative oxidase
CAT: catalase
ERK: extracellular signal regulated kinase
ETF: electron transfer flavoprotein
ETF:QO: electron transfer flavoprotein:ubiquinone oxidoreductase
GDPH: glycerol 3 phosphate dehydrogenase
GPX: glutathione peroxidase
GSH: tripeptide glutathione
G-3-P: glycerol-3-phosphate
MAPK/: mitogen activated protein kinase
NDA1: NADH dehydrogenase type II
OGDH: 2-oxoglutarate dehydrogenase complex
Q_i_: ubiquinone reduction centre
Q_o_: ubiquinol oxidation centre
ROS: reactive oxygen species
Sdh: succinate dehydrogenase
SOD: superoxide dismutase
TFAM: mitochondrial transcription factor A

## References

[1] Mitchell P. (1961) Coupling of phosphorylation to electron and hydrogen transfer by a chemi-osmotic type of mechanism. Nature 191, 144–148 10.1038/191144a0 13771349

[2] Dröse S. and Brandt U. (2012) Molecular mechanisms of superoxide production by the mitochondrial respiratory chain. In Mitochondrial Oxidative Phosphorylation (Kadenbach B., ed.), pp. 107–169, Springer 10.1007/978-1-4614-3573-0_6 22729857

[3] Guénebaut V., Schlitt A., Weiss H., Leonard K. and Friedrich T. (1998) Consistent structure between bacterial and mitochondrial NADH:ubiquinone oxidoreductase (complex I). J. Mol. Biol. 276, 105–112 10.1006/jmbi.1997.1518 9514725

[4] Baradaran R., Berrisford J.M., Minhas G.S. and Sazanov L. aa (2013) Crystal structure of the entire respiratory complex I. Nature 494, 443–448 10.1038/nature11871 23417064 PMC3672946

[5] Zickermann V., Wirth C., Nasiri H., Siegmund K., Schwalbe H., Hunte C. (2015) Mechanistic insight from the crystal structure of mitochondrial complex I. Science 5, 4–10 10.1126/science.1259859 25554780

[6] Zhu J., Vinothkumar K.R. and Hirst J. (2016) Structure of mammalian respiratory complex I. Nature 536, 354–358 10.1038/nature19095 27509854 PMC5027920

[7] Vinothkumar K.R., Zhu J. and Hirst J. (2014) Architecture of mammalian respiratory complex I. Nature 515, 80–84 10.1038/nature13686 25209663 PMC4224586

[8] Fiedorczuk K., Letts J.A., Degliesposti G., Kaszuba K., Skehel M. and Sazanov L.A. (2016) Atomic structure of the entire mammalian mitochondrial complex I. Nature 538, 406–410 10.1038/nature19794 27595392 PMC5164932

[9] Massoz S., Cardol P., González-Halphen D. and Remacle C. (2017) Mitochondrial bioenergetics pathways in Chlamydomonas. In Chlamydomonas: Molecular Genetics and Physiology (Hippler M., ed.), pp. 59–95, Springer

[10] Efremov R.G., Baradaran R. and Sazanov L. a. (2010) The architecture of respiratory complex I. Nature 465, 441–445 10.1038/nature09066 20505720

[11] Berrisford J.M., Baradaran R. and Sazanov L.A. (2016) Structure of bacterial respiratory complex I. Biochim. Biophys. Acta 1857, 892–901 10.1016/j.bbabio.2016.01.012 26807915

[12] Hirst J., Carroll J., Fearnley I.M., Shannon R.J. and Walker J.E. (2003) The nuclear encoded subunits of complex I from bovine heart mitochondria. Biochim. Biophys. Acta 1604, 135–150 10.1016/S0005-2728(03)00059-8 12837546

[13] Ohnishi T. (1998) Iron-sulfur clusters/semiquinones in complex I.. Biochim. Biophys. Acta 1364, 186–206 10.1016/S0005-2728(98)00027-9 9593887

[14] Brandt U. (2011) A two-state stabilization-change mechanism for proton-pumping complex I. Biochim. Biophys. Acta 1807, 1364–1369 10.1016/j.bbabio.2011.04.006 21565159

[15] Hummer G. and Wikström M. (2016) Molecular simulation and modeling of complex I. Biochim. Biophys. Acta 1857, 915–921 10.1016/j.bbabio.2016.01.005 26780586

[16] Stepanova A., Kahl A., Konrad C., Ten V., Starkov A.S. and Galkin A. (2017) Reverse electron transfer results in a loss of flavin from mitochondrial complex I: Potential mechanism for brain ischemia reperfusion injury. J. Cereb. Blood Flow Metab. 37, 3649–3658 10.1177/0271678X17730242 28914132 PMC5718331

[17] Wenz T., Hielscher R., Hellwig P., Schägger H., Richers S. and Hunte C. (2009) Role of phospholipids in respiratory cytochrome bc1 complex catalysis and supercomplex formation. Biochim. Biophys. Acta 1787, 609–616 10.1016/j.bbabio.2009.02.012 19254687

[18] Mitchell P. (1975) The protonmotive Q cycle: a general formulation. FEBS Lett. 59, 137–139 10.1016/0014-5793(75)80359-0 1227927

[19] Zhang Z., Huang L., Shulmeister V.M., Chi Y.-I., Kim K.K., Hung L.-W. (1998) Electron transfer by domain movement in cytochrome bc1. Nature 392, 677–684 10.1038/33612 9565029

[20] Hunte C. (2003) Protonmotive pathways and mechanisms in the cytochrome bc1 complex. FEBS Lett. 545, 39–46 10.1016/S0014-5793(03)00391-0 12788490

[21] Xia D., Esser L., Tang W.K., Zhou F., Zhou Y., Yu L. (2013) Structural analysis of cytochrome bc1 complexes: implications to the mechanism of function. Biochim. Biophys. Acta 1827, 1278–1294 10.1016/j.bbabio.2012.11.008 23201476 PMC3593749

[22] Bleier L. and Dröse S. (2013) Superoxide generation by complex III: From mechanistic rationales to functional consequences. Biochim. Biophys. Acta 1827, 1320–1331 10.1016/j.bbabio.2012.12.002 23269318

[23] Dröse S. (2013) Differential effects of complex II on mitochondrial ROS production and their relation to cardioprotective pre- and postconditioning. Biochim. Biophys. Acta 1827, 578–587 10.1016/j.bbabio.2013.01.004 23333272

[24] Quinlan C.L., Orr A.L., Perevoshchikova I. V, Treberg J.R., Ackrell B.A. and Brand M.D. (2012) Mitochondrial complex II can generate reactive oxygen species at high rates in both the forward and reverse reactions. J. Biol. Chem. 287, 27255–27264 10.1074/jbc.M112.374629 22689576 PMC3411067

[25] Wong H.S., Dighe P.A., Mezera V., Monternier P.A. and Brand M.D. (2017) Production of superoxide and hydrogen peroxide from specific mitochondrial sites under different bioenergetic conditions. J. Biol. Chem. 292, 16804–16809 10.1074/jbc.R117.789271 28842493 PMC5641882

[26] Brand M.D. (2016) Mitochondrial generation of superoxide and hydrogen peroxide as the source of mitochondrial redox signaling. Free Radic. Biol. Med. 100, 14–31 10.1016/j.freeradbiomed.2016.04.001 27085844

[27] Quinlan C.L., Goncalves R.L.S., Hey-Mogensen M., Yadava N., Bunik V.I. and Brand M.D. (2014) The 2-oxoacid dehydrogenase complexes in mitochondria can produce superoxide/hydrogen peroxide at much higher rates than complex I.. J. Biol. Chem. 289, 8312–8325 10.1074/jbc.M113.545301 24515115 PMC3961658

[28] Schertl P. and Braun H.-P. (2014) Respiratory electron transfer pathways in plant mitochondria. Front. Plant Sci. 5, 163 24808901 10.3389/fpls.2014.00163 PMC4010797

[29] Goncalves R.L.S., Bunik V.I. and Brand M.D. (2016) Production of superoxide/hydrogen peroxide by the mitochondrial 2-oxoadipate dehydrogenase complex. Free Radic. Biol. Med. 91, 247–255 10.1016/j.freeradbiomed.2015.12.020 26708453

[30] Shen W., Wei Y., Dauk M., Zheng Z. and Zou J. (2003) Identification of a mitochondrial glycerol-3-phosphate dehydrogenase from *Arabidopsis thaliana*: evidence for a mitochondrial glycerol-3-phosphate shuttle in plants. FEBS Lett. 536, 92–96 10.1016/S0014-5793(03)00033-4 12586344

[31] Mráček T., Drahota Z. and Houštěk J. (2013) The function and the role of the mitochondrial glycerol-3-phosphate dehydrogenase in mammalian tissues. Biochim. Biophys. Acta 1827, 401–410 10.1016/j.bbabio.2012.11.014 23220394

[32] Mráček T., Holzerová E., Drahota Z., Kovářová N., Vrbacký M., Ješina P. (2014) ROS generation and multiple forms of mammalian mitochondrial glycerol-3-phosphate dehydrogenase. Biochim. Biophys. Acta 1837, 98–111 10.1016/j.bbabio.2013.08.007 23999537

[33] Orr A.L., Quinlan C.L., Perevoshchikova I. V. and Brand M.D. (2012) A refined analysis of superoxide production by mitochondrial sn-glycerol 3-phosphate dehydrogenase. J. Biol. Chem. 287, 42921–42935 10.1074/jbc.M112.397828 23124204 PMC3522288

[34] Hey-Mogensen M., Goncalves R.L.S., Orr A.L. and Brand M.D. (2014) Production of superoxide/H2O2 by dihydroorotate dehydrogenase in rat skeletal muscle mitochondria. Free Radic. Biol. Med. 72, 149–155 10.1016/j.freeradbiomed.2014.04.007 24746616

[35] Watmough N.J. and Frerman F.E. (2010) The electron transfer flavoprotein: Ubiquinone oxidoreductases. Biochim. Biophys. Acta 1797, 1910–1916 10.1016/j.bbabio.2010.10.007 20937244

[36] Ayala A., Munoz M.F. and Arguelles S. (2014) Lipid peroxidation: production, metabolism, and signaling mechanisms of malondialdehyde and 4-hydroxy-2-nonenal. Oxid. Med. Cell Longev. 2014, 360438 10.1155/2014/360438 24999379 PMC4066722

[37] Adam-Vizi V. (2005) Production of reactive oxygen species in brain mitochondria: contribution by electron transport chain and non-electron transport chain sources. Antioxid. Redox Signal. 7, 1140–1149 10.1089/ars.2005.7.1140 16115017

[38] Mittler R., Vanderauwera S., Suzuki N., Miller G., Tognetti V.B., Vandepoele K (2011) ROS signaling: the new wave? Trends Plant Sci. 16, 300–309 10.1016/j.tplants.2011.03.007 21482172

[39] Aon M.A., Cortassa S. and O’Rourke B. (2010) Redox-optimized ROS balance: a unifying hypothesis. Biochim. Biophys. Acta 1797, 865–877 10.1016/j.bbabio.2010.02.016 20175987 PMC2891851

[40] Pozniakovsky A.I., Knorre D.A., Markova O. V, Hyman A.A., Skulachev V.P. and Severin F.F. (2005) Role of mitochondria in the pheromone- and amiodarone-induced programmed death of yeast. J. Cell Biol. 168, 257–269 10.1083/jcb.200408145 15657396 PMC2171581

[41] Finkel T. and Holbrook N.J. (2000) Oxidants, oxidative stress and the biology of ageing. Nature 408, 239–247 10.1038/35041687 11089981

[42] Squarcina A., Sorarù A., Carraro M., Geremia S., Morosinotto T. and Bonchio M. (2017) Merged heme and non-heme manganese cofactors for a dual antioxidant surveillance in photosynthetic organisms. ACS Catal. 7, 1971–1976 10.1021/acscatal.7b00004

[43] Suzuki Y.J., Carini M. and Butterfield D.A. (2010) Protein carbonylation. Antioxid. Redox Signal. 12, 323–325 10.1089/ars.2009.2887 19743917 PMC2821144

[44] Browne R.W. and Armstrong D. (2000) HPLC analysis of lipid-derived polyunsaturated fatty acid peroxidation products in oxidatively modified human plasma. Clin. Chem. 46, 829–836 10839772

[45] Schneider C., Boeglin W.E., Yin H., Porter N.A. and Brash A.R. (2008) Intermolecular peroxyl radical reactions during autoxidation of hydroxy and hydroperoxy arachidonic acids generate a novel series of epoxidized products. Chem. Res. Toxicol. 21, 895–903 10.1021/tx700357u 18324788

[46] Bielski B.H., Arudi R.L. and Sutherland M.W. (1983) A study of the reactivity of HO_2_/O_2_- with unsaturated fatty acids. J. Biol. Chem. 258, 4759–4761 6833274

[47] Birben E., Sahiner U.M., Sackesen C., Erzurum S. and Kalayci O. (2012) Oxidative stress and antioxidant defense. World Allergy Organ. J. 5, 9–19 10.1097/WOX.0b013e3182439613 23268465 PMC3488923

[48] Cooke M.S., Evans M.D., Dizdaroglu M. and Lunec J. (2003) Oxidative DNA damage: mechanisms, mutation, and disease. FASEB J. 17, 1195–1214 10.1096/fj.02-0752rev 12832285

[49] Sheng Y., Abreu I.A., Cabelli D.E., Maroney M.J., Miller A.F., Teixeira M. (2014) Superoxide dismutases and superoxide reductases. Chem. Rev. 114, 3854–3918 10.1021/cr4005296 24684599 PMC4317059

[50] Fridovich I. (1995) Superoxide radical and superoxide dismutases. Annu. Rev. Biochem. 64, 97–112 10.1146/annurev.bi.64.070195.000525 7574505

[51] Youn H.D., Kim E.J., Roe J.H., Hah Y.C. and Kang S.O. (1996) A novel nickel-containing superoxide dismutase from *Streptomyces* spp. Biochem. J. 318, 889–896 10.1042/bj3180889 8836134 PMC1217701

[52] Kowaltowski A.J., de Souza-Pinto N.C., Castilho R.F. and Vercesi A.E. (2009) Mitochondria and reactive oxygen species. Free Radic. Biol. Med. 47, 333–343 10.1016/j.freeradbiomed.2009.05.004 19427899

[53] Wolfe-Simon F., Starovoytov V., Reinfelder J.R., Schofield O. and Falkowski P.G. (2006) Localization and role of manganese superoxide dismutase in a marine diatom. Plant Physiol. 142, 1701–1709 10.1104/pp.106.088963 17056755 PMC1676035

[54] Ueda M., Kinoshita H., Maeda S.I., Zou W. and Tanaka A. (2003) Structure-function study of the amino-terminal stretch of the catalase subunit molecule in oligomerization, heme binding, and activity expression. Appl. Microbiol. Biotechnol. 61, 488–494 10.1007/s00253-003-1251-5 12764563

[55] Gill S.S. and Tuteja N. (2010) Reactive oxygen species and antioxidant machinery in abiotic stress tolerance in crop plants. Plant Physiol. Biochem. 48, 909–930 10.1016/j.plaphy.2010.08.016 20870416

[56] Heazlewood J.L., Tonti-Filippini J.S., Gout A.M., Day D.A., Whelan J. and Millar A.H. (2004) Experimental analysis of the arabidopsis mitochondrial proteome highlights signaling and regulatory components, provides assessment of targeting prediction programs, and indicates plant-specific mitochondrial proteins. Plant Cell 16, 241–256 10.1105/tpc.016055 14671022 PMC301408

[57] Petrova V.Y., Drescher D., Kujumdzieva A. V and Schmitt M.J. (2004) Dual targeting of yeast catalase A to peroxisomes and mitochondria. Biochem. J. 380, 393–400 10.1042/bj20040042 14998369 PMC1224190

[58] Mhamdi A., Queval G., Chaouch S., Vanderauwera S., Van Breusegem F. and Noctor G. (2010) Catalase function in plants: a focus on *Arabidopsis* mutants as stress-mimic models. J. Exp. Bot. 61, 4197–4220 10.1093/jxb/erq282 20876333

[59] Merchant S.S., Prochnik S.E., Vallon O., Harris E.H., Karpowicz S.J., Witman G.B. (2007) The *Chlamydomonas* genome reveals the evolution of key animal and plant functions. Science 318, 245–250 10.1126/science.1143609 17932292 PMC2875087

[60] Kato J., Yamahara T., Tanaka K., Takio S. and Satoh T. (1997) Characterization of catalase from green algae *Chlamydomonas reinhardtii*. J. Plant Physiol. 151, 262–268 10.1016/S0176-1617(97)80251-9

[61] Atteia A., Adrait A., Brugire S., Tardif M., Van Lis R., Deusch O. (2009) A proteomic survey of *Chlamydomonas reinhardtii* mitochondria sheds new light on the metabolic plasticity of the organelle and on the nature of the α-proteobacterial mitochondrial ancestor. Mol. Biol. Evol. 26, 1533–1548 10.1093/molbev/msp068 19349646

[62] Tardif M., Atteia A., Specht M., Cogne G., Rolland N., Brugière S. (2012) Predalgo: a new subcellular localization prediction tool dedicated to green algae. Mol. Biol. Evol. 29, 3625–3639 10.1093/molbev/mss178 22826458

[63] Reumann S., Chowdhary G. and Lingner T. (2016) Characterization, prediction and evolution of plant peroxisomal targeting signals type 1 (PTS1s). Biochim. Biophys. Acta 1863, 790–803 10.1016/j.bbamcr.2016.01.001 26772785

[64] Mittler R. (2002) Oxidative stress, antioxidants and stress tolerance. Trends Plant Sci. 7, 405–410 10.1016/S1360-1385(02)02312-9 12234732

[65] Mittler R., Vanderauwera S., Gollery M. and Van Breusegem F. (2004) Reactive oxygen gene network of plants. Trends Plant Sci. 9, 490–498 10.1016/j.tplants.2004.08.009 15465684

[66] Smirnoff N. (2018) Ascorbic acid metabolism and functions: a comparison of plants and mammals. Free Radic Biol Med., 10.1016/j.freeradbiomed.2018.03.033 PMC6191929 29567393

[67] Brigelius-Flohé R. and Maiorino M. (2013) Glutathione peroxidases. Biochim. Biophys. Acta 1830, 3289–3303 10.1016/j.bbagen.2012.11.020 23201771

[68] Miranda-Vizuete A., Damdimopoulos A.E. and Spyrou G. (2000) The mitochondrial thioredoxin system. Antioxid. Redox Signal. 2, 801–810 10.1089/ars.2000.2.4-801 11213484

[69] Guerrero-Castillo S., Cabrera-Orefice A., Vázquez-Acevedo M., González-Halphen D. and Uribe-Carvajal S. (2012) During the stationary growth phase, *Yarrowia lipolytica* prevents the overproduction of reactive oxygen species by activating an uncoupled mitochondrial respiratory pathway. Biochim. Biophys. Acta 1817, 353–362 10.1016/j.bbabio.2011.11.007 22138628

[70] Saha B., Borovskii G. and Panda S.K. (2016) Alternative oxidase and plant stress tolerance. Plant Signal. Behav. 11, e1256530 10.1080/15592324.2016.1256530 27830987 PMC5225930

[71] Feng Y., Li W., Li J., Wang J., Ge J., Xu D. (2012) Structural insight into the type-II mitochondrial NADH dehydrogenases. Nature 491, 478–482 10.1038/nature11541 23086143

[72] Lecler R., Vigeolas H., Emonds-Alt B., Cardol P. and Remacle C. (2012) Characterization of an internal type-II NADH dehydrogenase from chlamydomonas reinhardtii mitochondria. Curr. Genet. 58 205–216 22814755 10.1007/s00294-012-0378-2

[73] Siedow J.N. and Umbach A.L. (2000) The mitochondrial cyanide-resistant oxidase: Structural conservation amid regulatory diversity. Biochim. Biophys. Acta 1459, 432–439 10.1016/S0005-2728(00)00181-X 11004460

[74] Del-Saz N.F., Ribas-Carbo M., McDonald A.E., Lambers H., Fernie A.R. and Florez-Sarasa I. (2017) An *in vivo* perspective of the role(s) of the alternative oxidase pathway. Trends Plant Sci. 23, 206–219 10.1016/j.tplants.2017.11.006 29269217

[75] Skovsen E., Snyder J.W., Lambert J.D. and Ogilby P.R. (2005) Lifetime and diffusion of singlet oxygen in a cell. J. Phys. Chem. B 109, 8570–8573 10.1021/jp051163i 16852012

[76] Wood Z.A., Poole L.B. and Karplus P.A. (2003) Peroxiredoxin evolution and the regulation of hydrogen peroxide signaling. Science 300, 650–653 10.1126/science.1080405 12714747

[77] Allen R.G. and Tresini M. (2000) Oxidative stress and gene regulation. Free Radic. Biol. Med. 28, 463–499 10.1016/S0891-5849(99)00242-7 10699758

[78] Huang J., Willems P., Van Breusegem F. and Messens J. (2018) Pathways crossing mammalian and plant sulfenomic landscapes. Free Radic. Biol. Med. 122, 193–201 10.1016/j.freeradbiomed.2018.02.012 29476921

[79] Reczek C.R. and Chandel N.S. (2015) ROS-dependent signal transduction. Curr. Opin. Cell Biol. 33, 8–13 10.1016/j.ceb.2014.09.010 25305438 PMC4380867

[80] Schieber M. and Chandel N.S. (2014) ROS function in redox signaling and oxidative stress. Curr. Biol. 24, R453–R462 10.1016/j.cub.2014.03.034 24845678 PMC4055301

[81] Truong T.H. and Carroll K.S. (2013) Redox regulation of protein kinases. Crit. Rev. Biochem. Mol. Biol. 48, 332–356 10.3109/10409238.2013.790873 23639002 PMC4358782

[82] Roos G. and Messens J. (2011) Protein sulfenic acid formation: from cellular damage to redox regulation. Free Radic. Biol. Med. 51, 314–326 10.1016/j.freeradbiomed.2011.04.031 21605662

[83] Biteau B., Labarre J. and Toledano M.B. (2003) ATP-dependent reduction of cysteine-sulphinic acid by S. cerevisiae sulphiredoxin. Nature 425, 980–984 10.1038/nature02075 14586471

[84] Jonsson T.J., Murray M.S., Johnson L.C. and Lowther W.T. (2008) Reduction of cysteine sulfinic acid in peroxiredoxin by sulfiredoxin proceeds directly through a sulfinic phosphoryl ester intermediate. J. Biol. Chem. 283, 23846–23851 10.1074/jbc.M803244200 18579529 PMC2527103

[85] Rey P., Becuwe N., Barrault M.B., Rumeau D., Havaux M., Biteau B. (2007) The *Arabidopsis thaliana* sulfiredoxin is a plastidic cysteine-sulfinic acid reductase involved in the photooxidative stress response. Plant J. 49, 505–514 10.1111/j.1365-313X.2006.02969.x 17217469

[86] Jonsson T.J., Tsang A.W., Lowther W.T. and Furdui C.M. (2008) Identification of intact protein thiosulfinate intermediate in the reduction of cysteine sulfinic acid in peroxiredoxin by human sulfiredoxin. J. Biol. Chem. 283, 22890–22894 10.1074/jbc.C800124200 18593714 PMC2517003

[87] Dixon D.P., Skipsey M., Grundy N.M. and Edwards R. (2005) Stress-induced protein S-glutathionylation in *Arabidopsis*. Plant Physiol. 138, 2233–2244 10.1104/pp.104.058917 16055689 PMC1183410

[88] Liao X. and Butow R.A. (1993) RTG1 and RTG2: two yeast genes required for a novel path of communication from mitochondria to the nucleus. Cell 72, 61–71 10.1016/0092-8674(93)90050-Z 8422683

[89] Guha M. and Avadhani N.G. (2013) Mitochondrial retrograde signaling at the crossroads of tumor bioenergetics, genetics and epigenetics. Mitochondrion 13, 577–591 10.1016/j.mito.2013.08.007 24004957 PMC3832239

[90] Starkov A.A. (2008) The role of mitochondria in reactive oxygen species metabolism and signaling. Ann. N.Y. Acad. Sci. 1147, 37–52 10.1196/annals.1427.015 19076429 PMC2869479

[91] Gordeeva A. V, Zvyagilskaya R.A. and Labas Y.A. (2003) Cross-talk between reactive oxygen species and calcium in living cells. Biochem 68, 1077–1080 14616077 10.1023/a:1026398310003

[92] Adam-Vizi V. and Starkov A.A. (2010) Calcium and mitochondrial reactive oxygen species generation: how to read the facts. J. Alzheimers Dis. 20 (Suppl. 2), S413–S426 10.3233/JAD-2010-100465 20421693 PMC3056350

[93] Zima A. V and Blatter L.A. (2006) Redox regulation of cardiac calcium channels and transporters. Cardiovasc. Res. 71, 310–321 10.1016/j.cardiores.2006.02.019 16581043

[94] Brookes P.S., Yoon Y., Robotham J.L., Anders M.W. and Sheu S.S. (2004) Calcium, ATP, and ROS: a mitochondrial love-hate triangle. Am. J. Physiol. Cell Physiol. 287, C817–C833 10.1152/ajpcell.00139.2004 15355853

[95] Carden T., Singh B., Mooga V., Bajpai P. and Singh K.K. (2017) Epigenetic modification of miR-663 controls mitochondria-to-nucleus retrograde signaling and tumor progression. J. Biol. Chem. 292, 20694–20706 10.1074/jbc.M117.797001 29066618 PMC5733605

[96] Acín-Pérez R., Carrascoso I., Baixauli F., Roche-Molina M., Latorre-Pellicer A., Fernández-Silva P. (2014) ROS-triggered phosphorylation of complex II by Fgr kinase regulates cellular adaptation to fuel use. Cell Metab. 19, 1020–1033 10.1016/j.cmet.2014.04.015 24856931 PMC4274740

[97] Bleier L., Wittig I., Heide H., Steger M., Brandt U. and Dröse S. (2015) Generator-specific targets of mitochondrial reactive oxygen species. Free Radic. Biol. Med. 78, 1–10 10.1016/j.freeradbiomed.2014.10.511 25451644

[98] Nadtochiy S.M., Baker P.R., Freeman B.A. and Brookes P.S. (2009) Mitochondrial nitroalkene formation and mild uncoupling in ischaemic preconditioning: implications for cardioprotection. Cardiovasc. Res. 82, 333–340 10.1093/cvr/cvn323 19050010 PMC2675927

[99] Schopfer F.J., Batthyany C., Baker P.R., Bonacci G., Cole M.P., Rudolph V. (2009) Detection and quantification of protein adduction by electrophilic fatty acids: mitochondrial generation of fatty acid nitroalkene derivatives. Free Radic. Biol. Med. 46, 1250–1259 10.1016/j.freeradbiomed.2008.12.025 19353781 PMC3144282

[100] Koenitzer J.R. and Freeman B.A. (2010) Redox signaling in inflammation: interactions of endogenous electrophiles and mitochondria in cardiovascular disease. Ann. N.Y. Acad. Sci. 1203, 45–52 10.1111/j.1749-6632.2010.05559.x 20716282 PMC4106461

[101] Frohnert B.I. and Bernlohr D.A. (2013) Protein carbonylation, mitochondrial dysfunction, and insulin resistance. Adv. Nutr. 4, 157–163 10.3945/an.112.003319 23493532 PMC3649096

[102] Curtis J.M., Hahn W.S., Stone M.D., Inda J.J., Droullard D.J., Kuzmicic J.P. (2012) Protein carbonylation and adipocyte mitochondrial function. J. Biol. Chem. 287, 32967–32980 10.1074/jbc.M112.400663 22822087 PMC3463318

[103] Bourges I., Horan S. and Meunier B. (2005) Effect of inhibition of the bc1 complex on gene expression profile in yeast. J. Biol. Chem. 280, 29743–29749 10.1074/jbc.M505915200 15967791

[104] Delaunay A., Isnard A.D. and Toledano M.B. (2000) H_2_O_2_ sensing through oxidation of the Yap1 transcription factor. EMBO J. 19, 5157–5166 10.1093/emboj/19.19.5157 11013218 PMC302088

[105] Bersweiler A., D’Autreaux B., Mazon H., Kriznik A., Belli G., Delaunay-Moisan A. (2017) A scaffold protein that chaperones a cysteine-sulfenic acid in H_2_O_2_ signaling. Nat. Chem. Biol. 13, 909–915 10.1038/nchembio.2412 28628095

[106] Knorre D., Sokolov S., Zyrina A. and Severin F. (2016) How do yeast sense mitochondrial dysfunction? Microb. Cell 3, 532–539 10.15698/mic2016.11.537 28357322 PMC5349209

[107] Woo D.K., Green P.D., Santos J.H., D’Souza A.D., Walther Z., Martin W.D. (2012) Mitochondrial genome instability and ROS enhance intestinal tumorigenesis in APC(Min/+) mice. Am. J. Pathol. 180, 24–31 10.1016/j.ajpath.2011.10.003 22056359 PMC3338350

[108] Singh R.K., Srivastava A., Kalaiarasan P., Manvati S., Chopra R. and Bamezai R.N. (2014) mtDNA germ line variation mediated ROS generates retrograde signaling and induces pro-cancerous metabolic features. Sci. Rep. 4, 6571 10.1038/srep06571 25300428 PMC4192639

[109] Ogrunc M., Di Micco R., Liontos M., Bombardelli L., Mione M., Fumagalli M. (2014) Oncogene-induced reactive oxygen species fuel hyperproliferation and DNA damage response activation. Cell Death Differ. 21, 998–1012 10.1038/cdd.2014.16 24583638 PMC4013514

[110] Zhang L., Zhou L., Du J., Li M., Qian C., Cheng Y. (2014) Induction of apoptosis in human multiple myeloma cell lines by ebselen via enhancing the endogenous reactive oxygen species production. Biomed. Res. Int. 2014, 696107 24587987 10.1155/2014/696107 PMC3921973

[111] Wenzel P., Schuhmacher S., Kienhofer J., Muller J., Hortmann M., Oelze M. (2008) Manganese superoxide dismutase and aldehyde dehydrogenase deficiency increase mitochondrial oxidative stress and aggravate age-dependent vascular dysfunction. Cardiovasc. Res. 80, 280–289 10.1093/cvr/cvn182 18596060 PMC3937602

[112] Ekstrand M.I., Falkenberg M., Rantanen A., Park C.B., Gaspari M., Hultenby K. (2004) Mitochondrial transcription factor A regulates mtDNA copy number in mammals. Hum. Mol. Genet. 13, 935–944 10.1093/hmg/ddh109 15016765

[113] Kunkel G.H., Chaturvedi P. and Tyagi S.C. (2016) Mitochondrial pathways to cardiac recovery: TFAM. Heart Fail. Rev. 21, 499–517 10.1007/s10741-016-9561-8 27166683 PMC4985491

[114] Wang Y., Berkowitz O., Selinski J., Xu Y., Hartmann A. and Whelan J. (2018) Stress responsive mitochondrial proteins in *Arabidopsis thaliana*. Free Radic. Biol. Med. 10.1016/j.freeradbiomed.2018.03.031 29555593

[115] Fromm S., Senkler J., Eubel H., Peterhänsel C. and Braun H.P. (2016) Life without complex I: proteome analyses of an *Arabidopsis* mutant lacking the mitochondrial NADH dehydrogenase complex. J. Exp. Bot. 67, 3079–3093 10.1093/jxb/erw165 27122571 PMC4867900

[116] Wang Y., Lyu W., Berkowitz O., Radomiljac J.D., Law S.R., Murcha M.W. (2016) Inactivation of mitochondrial complex I induces the expression of a twin cysteine protein that targets and affects cytosolic, chloroplastidic and mitochondrial function. Mol. Plant 9, 696–710 10.1016/j.molp.2016.01.009 26829715

[117] Gleason C., Huang S., Thatcher L.F., Foley R.C., Anderson C.R., Carroll A.J. (2011) Mitochondrial complex II has a key role in mitochondrial-derived reactive oxygen species influence on plant stress gene regulation and defense. Proc. Natl. Acad. Sci. U.S.A. 108, 10768–10773 10.1073/pnas.1016060108 21670306 PMC3127871

[118] Ng S., Ivanova A., Duncan O., Law S.R., Van Aken O., De Clercq I. (2013) A membrane-bound NAC transcription factor, ANAC017, mediates mitochondrial retrograde signaling in *Arabidopsis*. Plant Cell 25, 3450–3471 10.1105/tpc.113.113985 24045017 PMC3809543

[119] Larosa V., Coosemans N., Motte P., Bonnefoy N. and Remacle C. (2012) Reconstruction of a human mitochondrial complex i mutation in the unicellular green alga *Chlamydomonas*. Plant J. 70, 759–768 10.1111/j.1365-313X.2012.04912.x 22268373

[120] Massoz S., Larosa V., Plancke C., Lapaille M., Bailleul B., Pirotte D. (2014) Inactivation of genes coding for mitochondrial Nd7 and Nd9 complex I subunits in *Chlamydomonas reinhardtii*. Impact of complex I loss on respiration and energetic metabolism. Mitochondrion 19, 365–374 10.1016/j.mito.2013.11.004 24316185

[121] Remacle C., Coosemans N., Jans F., Hanikenne M., Motte P. and Cardol P. (2010) Knock-down of the COX3 and COX17 gene expression of cytochrome c oxidase in the unicellular green alga *Chlamydomonas reinhardtii*. Plant Mol. Biol. 74, 223–233 20700628 10.1007/s11103-010-9668-6

[122] Roach T., Na C.S. and Krieger-Liszkay A. (2015) High light-induced hydrogen peroxide production in *Chlamydomonas reinhardtii* is increased by high CO2 availability. Plant J. 81, 759–766 10.1111/tpj.12768 25619314

[123] Murik O., Elboher A. and Kaplan A. (2014) Dehydroascorbate: a possible surveillance molecule of oxidative stress and programmed cell death in the green alga *Chlamydomonas reinhardtii*. New Phytol. 202, 471–484 10.1111/nph.12649 24345283

